# Characterization of tau propagation pattern and cascading hypometabolism from functional connectivity in Alzheimer's disease

**DOI:** 10.1002/hbm.26689

**Published:** 2024-05-04

**Authors:** Min Wang, Jiaying Lu, Ying Zhang, Qi Zhang, Luyao Wang, Ping Wu, Matthias Brendel, Axel Rominger, Kuangyu Shi, Qianhua Zhao, Jiehui Jiang, Chuantao Zuo

**Affiliations:** ^1^ School of Life Sciences Shanghai University Shanghai China; ^2^ Department of Nuclear Medicine & PET Center, Huashan Hospital Fudan University Shanghai China; ^3^ National Clinical Research Center for Aging and Medicine, Huashan Hospital Fudan University Shanghai China; ^4^ National Center for Neurological Disorders, Huashan Hospital Fudan University Shanghai China; ^5^ Department of Nuclear Medicine University of Munich Munich Germany; ^6^ Department of Nuclear Medicine, Inselspital Bern University Hospital, University of Bern Bern Switzerland; ^7^ Computer Aided Medical Procedures, School of Computation, Information and Technology Technical University of Munich Munich Germany; ^8^ Department of Neurology, Huashan Hospital Fudan University Shanghai China; ^9^ Human Phenome Institute Fudan University Shanghai China

**Keywords:** Alzheimer's disease, functional connectivity, glucose metabolism, PET imaging, tau pathology

## Abstract

Tau pathology and its spatial propagation in Alzheimer's disease (AD) play crucial roles in the neurodegenerative cascade leading to dementia. However, the underlying mechanisms linking tau spreading to glucose metabolism remain elusive. To address this, we aimed to examine the association between pathologic tau aggregation, functional connectivity, and cascading glucose metabolism and further explore the underlying interplay mechanisms. In this prospective cohort study, we enrolled 79 participants with ^18^F‐Florzolotau positron emission tomography (PET), ^18^F‐fluorodeoxyglucose PET, resting‐state functional, and anatomical magnetic resonance imaging (MRI) images in the hospital‐based Shanghai Memory Study. We employed generalized linear regression and correlation analyses to assess the associations between Florzolotau accumulation, functional connectivity, and glucose metabolism in whole‐brain and network‐specific manners. Causal mediation analysis was used to evaluate whether functional connectivity mediates the association between pathologic tau and cascading glucose metabolism. We examined 22 normal controls and 57 patients with AD. In the AD group, functional connectivity was associated with Florzolotau covariance (*β* = .837, *r* = 0.472, *p* < .001) and glucose covariance (*β* = 1.01, *r* = 0.499, *p* < .001). Brain regions with higher tau accumulation tend to be connected to other regions with high tau accumulation through functional connectivity or metabolic connectivity. Mediation analyses further suggest that functional connectivity partially modulates the influence of tau accumulation on downstream glucose metabolism (mediation proportion: 49.9%). Pathologic tau may affect functionally connected neurons directly, triggering downstream glucose metabolism changes. This study sheds light on the intricate relationship between tau pathology, functional connectivity, and downstream glucose metabolism, providing critical insights into AD pathophysiology and potential therapeutic targets.

## Graphical abstract

This study investigated the association between pathologic tau aggregation, functional connectivity, and cascading glucose metabolism and further explored the underlying interplay mechanisms. Pathologic tau may affect functionally connected neurons directly, triggering downstream glucose metabolism changes.

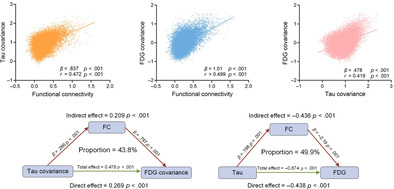

## 1 INTRODUCTION

Alzheimer's disease (AD) is characterized by the widespread extracellular deposition of amyloid‐β, intraneuronal tau neurofibrillary tangles, and neurodegeneration trajectories, which can be measured in vivo using positron emission tomography (PET) imaging (Alzheimer's Association Report, 2023; Jack Jr. et al., 2018; Kreisl et al., 2020). Converging AD findings suggest that the aggregation of pathological tau is followed by a complex cascade of downstream neurodegeneration or neuronal injury and contributions to cognitive decline (Boccalini et al., 2024; Boxer & Sperling, 2023; Kaufman et al., 2016; La Joie et al., 2020; Ossenkoppele et al., 2019). However, the interplay mechanisms by which tau neurofibrillary tangles spread spatially, leading to downstream pathological changes and clinical symptoms, are still a pressing issue in AD. The development of PET imaging biomarkers for tau and neurodegeneration offers an opportunity not only to visualize and quantify the pathophysiology in the living brains of AD, but also to investigate the interaction mechanisms of the tau spatial propagation and its cascading neurodegeneration (Lu et al., 2023; Schöll et al., 2016).

Extensive in vivo and in vitro studies have demonstrated that the distribution and spreading of pathologic tau may follow a prion‐like neuron‐to‐neuron transmission pattern, where brain regions carrying pathological tau are more strongly propagated along connected region through neuronal activity (Gibbons et al., 2019; Guo & Lee, 2011; Mudher et al., 2017; Zhou et al., 2012). In accordance with this concept, recent in vitro evidence suggests that injection of pathologic tau into transgenic mice brain triggers the spreading of tau toward synaptically connected regions rather than diffusion to spatially adjacent regions (Clavaguera et al., 2009; de Calignon et al., 2012). Moreover, a series of joint analyses between tau PET and resting‐state functional magnetic resonance imaging (fMRI) contribute significantly to our understanding of the association between pathologic tau aggregation and functional connectivity in AD and other tauopathies (Cope et al., 2018; Franzmeier et al., 2019, 2022; Franzmeier, Neitzel, et al., 2020; Schoonhoven et al., 2023; Schumacher et al., 2021). These findings demonstrate that functional connectivity or inter‐regional connectivity is thought to play a critical role in the distribution and propagation of tau, where higher inter‐regional tau covariance is strongly associated with higher functional connectivity between corresponding regions (Franzmeier et al., 2019, 2022; Franzmeier, Neitzel, et al., 2020; Schumacher et al., 2021). Additionally, higher functional connectivity of a target region with the tau epicenter is associated with higher tau aggregation levels in the target region (Cope et al., 2018; Franzmeier et al., 2019). Together, these findings advance the understanding of functional connectivity‐dependent patterns of pathologic tau spreading in the brain.

What remained unclear, however, is the downstream glucose metabolism mechanism underlying this facilitated tau spreading. Previous AD studies show that pathologic tau mediates direct toxic effects on neurons, thereby triggering widespread downstream changes in function and metabolism (Ballatore et al., 2007; Ittner & Götz, 2011; Ossenkoppele et al., 2016). This is supported by preclinical work, showing a potential causal relationship between tau pathology and the onset of neuronal dysfunction (Bischof et al., 2016; Rubinski et al., 2020; van Eimeren et al., 2017). As pathologic tau spreads, brain regions exhibiting stronger functional connectivity will accrue more pathologic tau aggregation, which may manifest as a more vulnerable trophic supply or hypometabolism (Chennu et al., 2017). While compelling findings support the connect‐based pattern of tau propagation and the link between tau pathology and glucose metabolism, respectively, the interaction mechanism and the role of cascading glucose metabolism in tau spreading are poorly understood and of pivotal clinical importance.

To address this pivotal question, we prospectively assessed and measured the associations between pathologic tau accumulation, functional connectivity, and cascading glucose metabolism using ^18^F‐Florzolotau PET imaging, fMRI, and ^18^F‐fluorodeoxyglucose (FDG) PET imaging obtained from patients with AD. The primary aim of this present observational study is to examine the brain architecture of tau spreading through the associations between tau covariance, functional connectivity, and metabolic covariance. We further employ mediation analysis to investigate whether the association between pathologic tau and metabolism changes can be explained by functional connectivity.

## 2 METHODS

### 2.1 Participants

All participants were recruited from the Memory Clinic of the Department of Neurology carried out at the Huashan Hospital (Fudan University, Shanghai, China), and this study was conducted as part of the hospital‐based Shanghai Memory Study (SMS) (Xiao et al., 2022). We enrolled 22 normal control (NC) subjects and 57 patients with AD, as evidenced by the clinical diagnosis of AD based on the National Institute of Neurological and Communicative Disorders and Stroke and the Alzheimer's Disease and Related Disorders Association (NINCDS‐ADRDA) criteria and a ^18^F‐AV‐45 (florbetapir) PET imaging assessment of positive amyloid pathology (Lu et al., 2023; McKhann et al., 1984). Eligibility criteria for NC subjects comprised the absence of cognitive complaints or concerns during the structured interview and a negative history of neurological or psychiatric disorders. AD patients underwent neuropsychological testing (Mini‐Mental State Examination [MMSE]; Clinical Dementia Rating‐Global Scale [CDR‐GS]) to assess global cognition. The detailed demographic and clinical information are given in Table 1.

**TABLE 1:** The clinical and demographic characteristics.

	NC	AD	*p*‐Value
Number	22	57	–
Age (years)	55.7 ± 8.8	64.9 ± 10.0	.0003^a^
Gender (*M*/*F*)	7/15	22/35	.578^b^
Education (years)	10.0 ± 4.5	10.3 ± 3.6	.786^a^
MMSE^d^	27.6 ± 1.5	20.3 ± 6.5	.0004^a^
CDR‐GS^d^	0 ± 0	1.07 ± 0.58	<.0001^c^

*Note*: Data are reported as means ± standard deviation.

Abbreviations: AD, Alzheimer's disease; CDR‐GS, Clinical Dementia Rating‐Global Scale; MMSE, Mini‐Mental State Examination; NC, normal control.

^a^ Independent student *t*‐test.

^b^ Chi‐square test.

^c^ Mann–Whitney *U* test.

^d^ Thirteen out of the 22 normal controls had detailed neuropsychological testing scores (MMSE and CDR‐SB).

### 2.2 Standard protocol approvals, registrations, and patient consents

All procedures performed in studies involving human participants were in accordance with the ethical standards of the institutional and/or national research committee and with the 1964 Declaration of Helsinki and its later amendments or comparable ethical standards. The data analysis and ethical permission of this study were approved by the Institutional Review Board of Huashan Hospital (clinical registration: 2000028864), and informed consent was obtained from all subjects after receiving a detailed explanation of the study procedures.

### 2.3 Image acquisition

All participants underwent ^18^F‐Florzolotau and ^18^F‐FDG PET, anatomical T1‐weighted magnetic resonance imaging (MRI), and fMRI scanning in Huashan Hospital. ^18^F‐Florzolotau (also known as ^18^F‐APN‐1607 or ^18^F‐PM‐PBB3) is a new‐generation tau radioligand with high affinity to both three‐ and four‐repeated tau (Tagai et al., 2021), and its tosylate precursor were provided by APRINOIA Therapeutics (Suzhou, China). All ^18^F‐Florzolotau (90–110 min post‐injection) and ^18^F‐FDG (60–70 min post‐injection) PET images were scanned on a Biograph mCT Flow PET/CT scanner (Siemens, Erlangen, Germany) after intravenous injection of ^18^F‐Florzolotau (370 mBq) or ^18^F‐FDG (185 mBq) on various study days. The detailed parameters can be found elsewhere (Liu et al., 2023; Lu, Wang, et al., 2023). All anatomical T1‐weighted MRI and fMRI were consecutively scanned on a 3.0‐T horizontal magnet scanner (Discovery MR750; GE Medical Systems, Milwaukee, WI) using the following parameters: echo time (TE), 3.2 ms; repetition time (TR), 8.2 ms; TI, 450 ms; flip angle, 12°; 25.6 cm field of view (FOV); acquisition matrix, 256 × 256 × 152; and voxel size, 1 × 1 × 1 mm. A total of 210 resting‐state fMRI images were also obtained using a 3D gradient echo planar image sequence in 3.0 mm isotropic voxel resolution with TE, 145 ms; TR, 8800 ms, flip angle, 77°; and 24 cm FOV. All PET and MRI scans for the subjects were conducted within a week of each other.

### 2.4 Image preprocessing

T1‐weighted MRI, ^18^F‐Florzolotau, and ^18^F‐FDG PET images of each participant were processed using Statistical Parametric Mapping 12 (SPM12; Wellcome Department of Imaging Neuroscience, Institute of Neurology, London, UK) implemented in MATLAB (version 2021b, MathWorks, Natick, MA). All PET frames were realigned, averaged, and coregistered onto their native space corresponding T1‐weighted MRI image. Individual T1‐weighted MRI image was then segmented into gray matter, white matter, and cerebrospinal fluid (CSF) tissue using the SPM unified segmentation pipeline. For ^18^F‐Florzolotau and ^18^F‐FDG PET images, we employed the voxel‐based Müller‐Gärtner method to perform partial volume effects correction (Gonzalez‐Escamilla et al., 2017). PET images were warped to Montreal Neurological Institute (MNI) standard space using the deformation parameters estimated during the MRI segmentation step. Subsequently, normalized PET images were smoothed with a Gaussian kernel at half maximum with an 8 mm full width. ^18^F‐Florzolotau and ^18^F‐FDG PET images were intensity normalized to the mean tracer uptake of the inferior cerebellar gray matter and cerebral cortical region to determine standardized uptake value ratio (SUVR) maps. Mean Florzolotau and FDG SUVR values were calculated for each participant using the Schaefer 200 cortical brain atlas (Schaefer et al., 2018).

Resting‐state fMRI images were processed using the Data Processing and Analysis for Brain Imaging toolbox (Yan et al., 2016). In brief, the first 10 volumes of the fMRI time series were removed to allow for the magnetization to reach a steady state. fMRI image was slice‐time and motion‐corrected, and coregistered to the corresponding T1‐weighted MRI image. We applied motion correction (i.e., realignment), regressed out the mean signal from the white matter and CSF, as well as the six motion parameters that were estimated during realignment (i.e., three translations and three rotations). We further removed the linear trend and applied band‐pass filtering with a 0.01–0.08 Hz frequency band. To remove the additional motion‐related artifacts, a 24‐parameter motion model (including six rigid‐body motion parameters, six temporal derivatives, and these terms squared) was used to perform scrubbing and remove the high‐motion frames. Last, the whole fMRI volumes were warped to MNI space using the deformation parameters and finally smoothed with 4 mm full width at half maximum Gaussian kernel.

### 2.5 Functional connectivity assessment

The preprocessed fMRI volumes were used to estimate functional connectivity for each participant. Individual fMRI data were then parcellated into 200 cortical regions of interest (ROIs) based on the Schaefer 200 brain atlas, and the mean time series from each brain region were extracted by averaging the signal across voxels (Schaefer et al., 2018). We estimated functional connectivity as Pearson correlations between all possible pairwise regions using the 200 ROI‐specific time series, yielding a 200 × 200 functional connectivity matrix for each participant. The pairwise correlation value was subsequently Fisher‐*Z* transformed to normalize the correlation matrix, and autocorrelations were set to zero. Lastly, group‐averaged functional connectivity matrices were calculated across individual functional connectivity for each group (NC and AD).

### 2.6 Tau and FDG covariance assessment

Similar to functional connectivity, Florzolotau and FDG PET covariance (i.e., metabolic connectivity) were estimated in an ROI‐based manner using the Schaefer 200 brain atlas. In brief, the mean Florzolotau and FDG within each of the 200 ROIs for each participant were extracted, yielding 200‐element vectors of Florzolotau aggregation and glucose activity per subject. These Florzolotau and FDG SUVR vectors were corrected between pairwise regions and further obtained the ROI‐to‐ROI Spearman correlation matrix. To avoid the estimation of PET covariance derived by extreme Florzolotau or FDG SUVR in specific regions or subjects, the Spearman correlation was used to measure the pairwise correlation value. We further obtained a single 200 × 200‐sized covariance in the Florzolotau or FDG matrix for each group (NC and AD). Similar to functional connectivity, the Florzolotau and FDG covariance matrices were Fisher‐*Z* transformed, and autocorrelations were set to zero. Additional covariance in Florzolotau or FDG matrix controlled for age, gender, and education was obtained using partial correlation.

### 2.7 Statistical analyses

Group differences between NC and AD in clinical characteristics were compared using an independent student *t*‐test for continuous variables, a chi‐squared test for categorical variables, and a Mann–Whitney *U* test for ordinal variables. To characterize the spatial PET distribution of Florzolotau accumulation and FDG metabolism, we calculated voxel‐wise Florzolotau and FDG SUVR maps for the NC and AD groups. We also measured Florzolotau accumulation and FDG metabolism in a network‐specific manner across seven canonical networks (Yeo et al., 2011). The uptake of tau and FDG for each network was measured by averaging the uptake of the parcels within the specific network parcellation. The seven functional networks included the dorsal attention network (DAN), default mode network (DMN), frontoparietal control network (FPCN), somatomotor network (Motor), ventral attention network (VAN), visual network (Vis), and limbic network (Limbic). The network‐specific group differences of Florzolotau and FDG uptake were measured using the two‐sample *t*‐test.

To investigate the association between functional connectivity and covariance of Florzolotau and FDG, we employed the generalized linear regression model and Pearson's correlation analysis to measure the association values using vectorized functional connectivity and vectorized PET covariance data. This association analysis was determined to assess whether regions with strong functional connectivity show higher covariance in tau pathology accumulation (or glucose metabolism dysfunction), indicative of transneuronal tau spread (or metabolic imbalance). Meanwhile, we also measured these associations separately across the seven canonical networks. To increase the statistical power of the association, we used 500 shuffled functional connectivity models and derived a null‐distribution of the *β* values that were used to compare the true *β* value using an exact test (Váša & Mišić, 2022). We further assessed whether the pathologic tau accumulation in a given seed ROI was associated with the Florzolotau uptake in closely connected regions of AD. We rank‐ordered all ROIs by Florzolotau accumulation level and then used the generalized linear regression model to regress the Florzolotau SUVR in the targeted regions onto the functional connectivity between the target ROIs and the given seed ROI. We repeated this association analysis across all 200 ROIs, yielding a series of *β*‐values. The above association analyses were further repeated while additionally controlling the regression model for Euclidean distance between each ROI pair to assess whether associations between functional connectivity and PET covariances were independent of distance. To measure whether higher Florzolotau SUVR in the seed region is related to higher connectivity‐related Florzolotau in target regions, we employed Pearson's correlation to calculate the relationship between the Florzolotau SUVR in a seed region and the corresponding *β*‐values for a given seed region. We also measured the *β*‐values for specific seed regions with maximum Florzolotau SUVR (i.e., hotspot) and minimum Florzolotau SUVR (i.e., coldspot). Again, we performed the same statistical analyses using the 500 shuffled functional connectivity to compare the true *β*‐value with a *β*‐value null‐distribution using an exact test. We repeated the above analyses to assess the relationships between Florzolotau SUVR and FDG covariance and between functional connectivity and FDG SUVR.

Lastly, we investigated whether the association between pathologic tau and metabolism changes can be explained by functional connectivity. The rationale is that if pathologic tau spreads as a function of functional connectivity, then regions with similar Florzolotau accumulation may be triggering downstream metabolism abnormality because of the direct toxic effects on neurons of pathologic tau. To test this, we employed mediation analyses to evaluate the association between Florzolotau and FDG‐mediated functional connectivity. The first equation regressed the mediator (functional connectivity) on the independent variable (Florzolotau covariance or Florzolotau SUVR). The second equation regressed the dependent variable (FDG covariance or FDG SUVR) on the independent variable. The third equation regressed the dependent variable on both the independent variable and the mediator variable. The success of the mediation effects should satisfy the following criteria simultaneously: (1) Florzolotau was associated with functional connectivity significantly; (2) Florzolotau was associated with FDG significantly; (3) functional connectivity was associated with FDG significantly; (4) the association between Florzolotau and FDG was reduced when functional connectivity (the mediator) was added in the regression model. To assess the change in degrees of glucose metabolism in AD, mean FDG *Z*‐scores of AD patients were calculated using the mean and standard deviation from the NC group as reference values. Age, gender, and education were controlled in the above mediation analyses. The mediation analyses were conducted on R software (version 3.5.1) using the mediate package. Two‐sided *p* values <.05 were considered significant.

## 3 RESULTS

### 3.1 Sample characteristics

Demographic and clinical information for 22 NC and 57 patients with AD are presented in Table 1. The AD patients were relatively older than the NC subjects, which was statistically significant (*p* = .0003). As expected, the neuropsychological scores of NC and AD differed significantly (*p* < .001), with lower MMSE scores and higher CDR‐GS scores in AD patients. There were no significant group differences for gender (*p* = .578) and years of education (*p* = .786).

### 3.2 Spatial distribution of Florzolotau and FDG in AD

**FIGURE 1:**
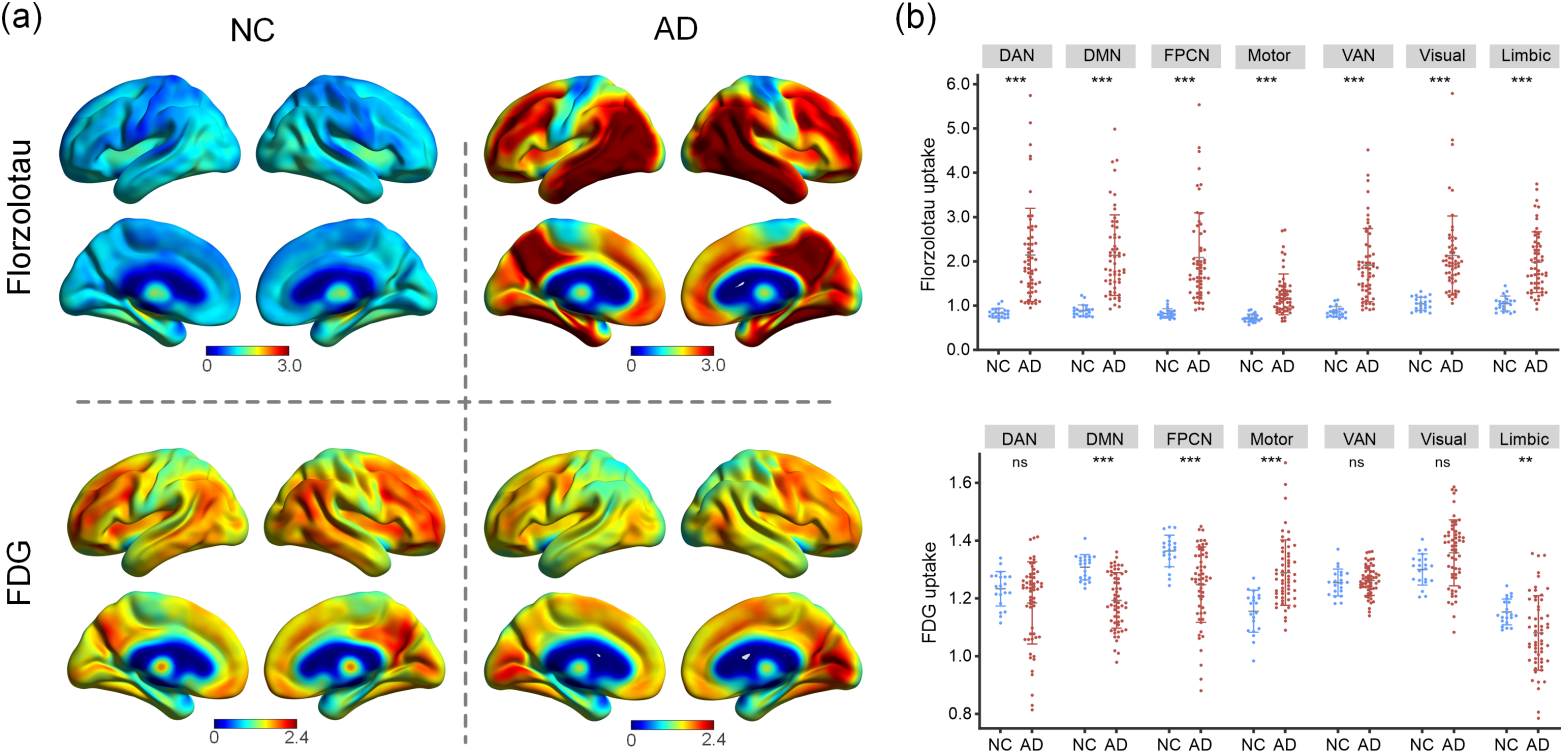
Voxel‐wise and region‐wise positron emission tomography (PET) patterns in each group. (a) Average PET distributions of Florzolotau and glucose metabolism for normal control (NC) and Alzheimer's disease (AD) groups. (b) Network‐specific Florzolotau and fluorodeoxyglucose (FDG) standardized uptake value ratio level for NC and AD group. DAN, dorsal attention network; DMN, default mode network; FPCN, frontoparietal control network; Limbic, limbic network; Motor, somatomotor network; VAN, ventral attention network; ns, not significant; ***p < .0001; **p < .001.

The spatial distribution patterns of Florzolotau binding and FDG metabolism are shown in Figure 1a. In line with previous AD typical Braak‐like findings, Florzolotau accumulation predominated in temporo‐parietal junction, precuneus, and posterior cingulate, widespread Florzolotau accumulation across frontal, occipital, parietal, and infero‐medial temporal cortices in the AD group were evaluated. Meanwhile, network‐specific Florzolotau accumulation in the AD group was maximal in the DAN region and moderate in all other major functional networks. There were significant Florzolotau accumulation differences across all functional networks between the NC and AD groups (all *p* < .001, Figure 1b). In AD, glucose hypometabolism manifested by FDG PET was found in the posterior cingulate, precuneus, and parietotemporal association cortices. Network‐specific metabolic analyses showed that hypometabolism predominated in DMN (*p* < .0001), followed by hypometabolism degree in FPCN (*p* < .0001) and Limbic (*p* < .001), whereas DAN, VAN, and Vis were mostly spared (*p* > .05).

### 3.3 Functional connectivity and Florzolotau and FDG covariance

**FIGURE 2:**
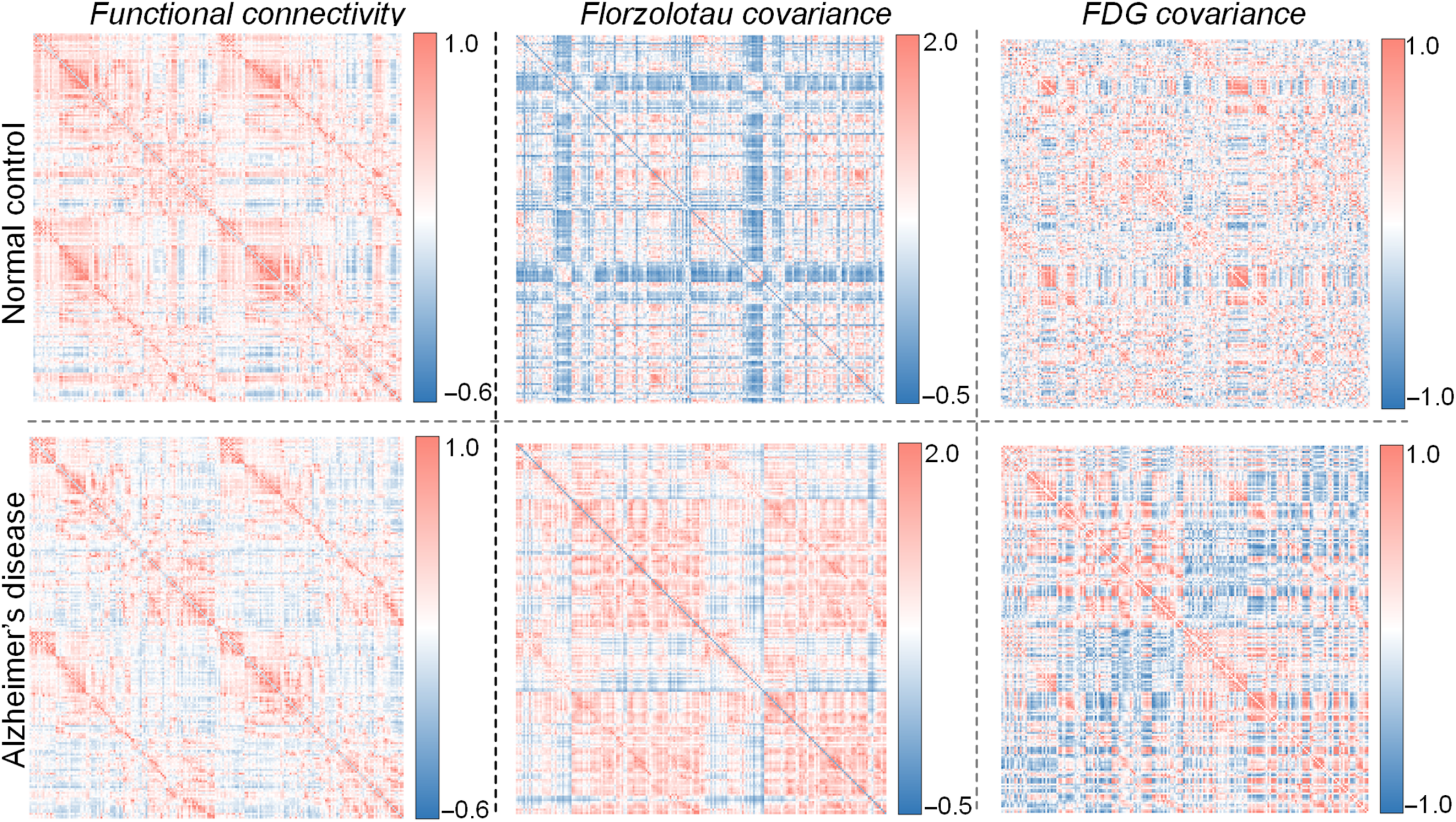
Average functional connectivity and covariance of Florzolotau and fluorodeoxyglucose (FDG) matrices in normal control and Alzheimer's disease groups. All correlation values in the 200 × 200 matrix were Fisher‐*Z* transformed.

To assess the relationship between functional connectivity and PET covariance, we calculated and obtained the average‐grouped functional connectivity, Florzolotau covariance, and FDG covariance across groups (Figure 2). The higher correlations in PET covariance characterize similar metabolic activity (pathologic tau accumulation or glucose metabolism) within pairwise regions. While one can make general observations from these matrices, such as a general reduction in between‐lobe functional connectivity in AD, a prominent enhancement in inter‐ and intra‐lobe Florzolotau covariance in AD, and a general reduction in between‐lobe FDG covariance in AD, the insights would be limited. Instead, spatial regression between functional connectivity and PET covariance allows us to evaluate whether functionally connected regions show similar PET metabolic activity and further manifest transneuronal pathologic tau spread. Because there was no significant pathologic tau accumulation in the NC group, the association analysis and subsequent assessments were conducted in the AD group.

**FIGURE 3:**
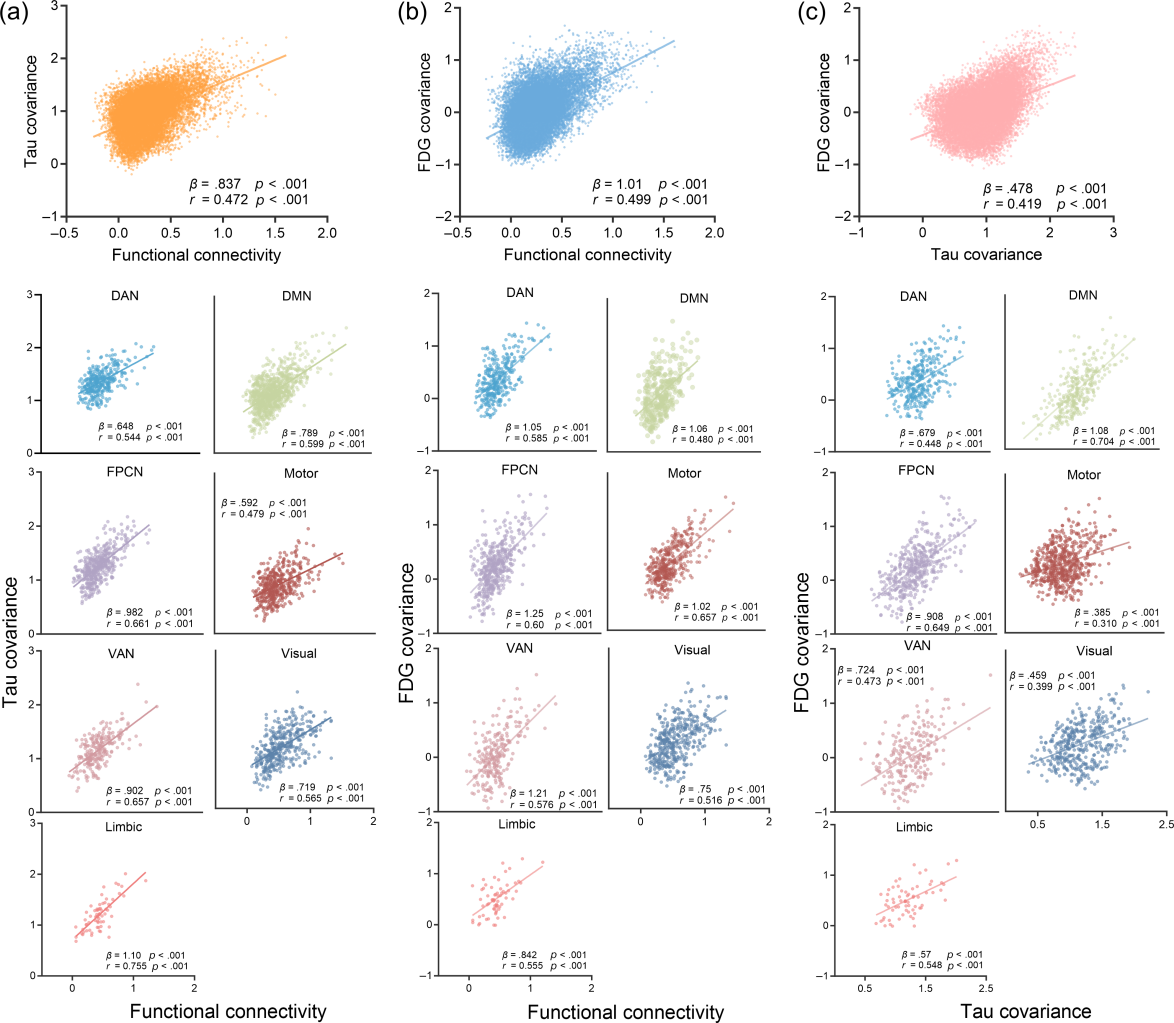
Associations between functional connectivity and positron emission tomography covariance. Scatterplots showing the associations between functional connectivity and Florzolotau covariance (a), fluorodeoxyglucose (FDG) covariance (b), and Florzolotau covariance and FDG covariance (c). DAN, dorsal attention network; DMN, default mode network; FPCN, frontoparietal control network; Limbic, limbic network; Motor, somatomotor network; VAN, ventral attention network.

Figure 3 shows the associations between functional connectivity and PET covariance (Florzolotau and FDG) in whole‐brain and network‐specific manners. There was a significant positive association between functional connectivity and Florzolotau covariance (*β* = .837, *p* < .001; *r* = 0.472, *p* < .001, Figure 3a). The 500 times null‐models obtained shuffled *β*‐value distributions (*β* = .028 ± .008), surviving exact test (*p* < .001) for comparing the true *β*‐value with null‐distribution derived *β*‐values. We also found significant positive associations between functional connectivity and Florzolotau covariance within all seven functional networks (range of *β* value = [.648–1.10], range of *r* value = [0.479–0.755], all *p* < .001). These results suggest that higher functional connectivity is associated with more similar Florzolotau accumulation in AD. There was a significant positive association between functional connectivity and FDG covariance (*β* = 1.01, *p* < .001; *r* = 0.499, *p* < .001, Figure 3b). The null‐models obtained shuffled *β*‐value distributions (*β* = −.008 ± .01), surviving exact test (*p* < .001). We also found significant positive associations between functional connectivity and FDG covariance within all seven functional networks (range of *β* value = [.75–1.25], range of *r* value = [0.48–0.657], all *p* < .001). These results suggest that higher functional connectivity is also associated with a more similar glucose metabolism in AD. Furthermore, we found a significant positive association between Florzolotau covariance and FDG covariance (*β* = .478, *p* < .001; *r* = 0.419, *p* < .001, Figure 3c). The null‐models obtained shuffled *β*‐value distributions (*β* = .089 ± .003), surviving exact test (*p* < .001). We also found significant positive associations between Florzolotau covariance and FDG covariance within all seven functional networks (range of *β* value = [.385–1.08], range of *r* value = [0.31–0.704], all *p* < .001). The above associations remained consistent when controlling the assessment of PET covariances for age, sex, and education (Supporting Information S1). These results suggest that similar Florzolotau accumulation is also associated with a more similar glucose metabolism in AD. The significant associations between functional connectivity and covariance of Florzolotau and FDG may indicate the interaction mechanism underlying tau spreading.

### 3.4 Associations between functional connectivity and Florzolotau and FDG‐PET uptake

**FIGURE 4:**
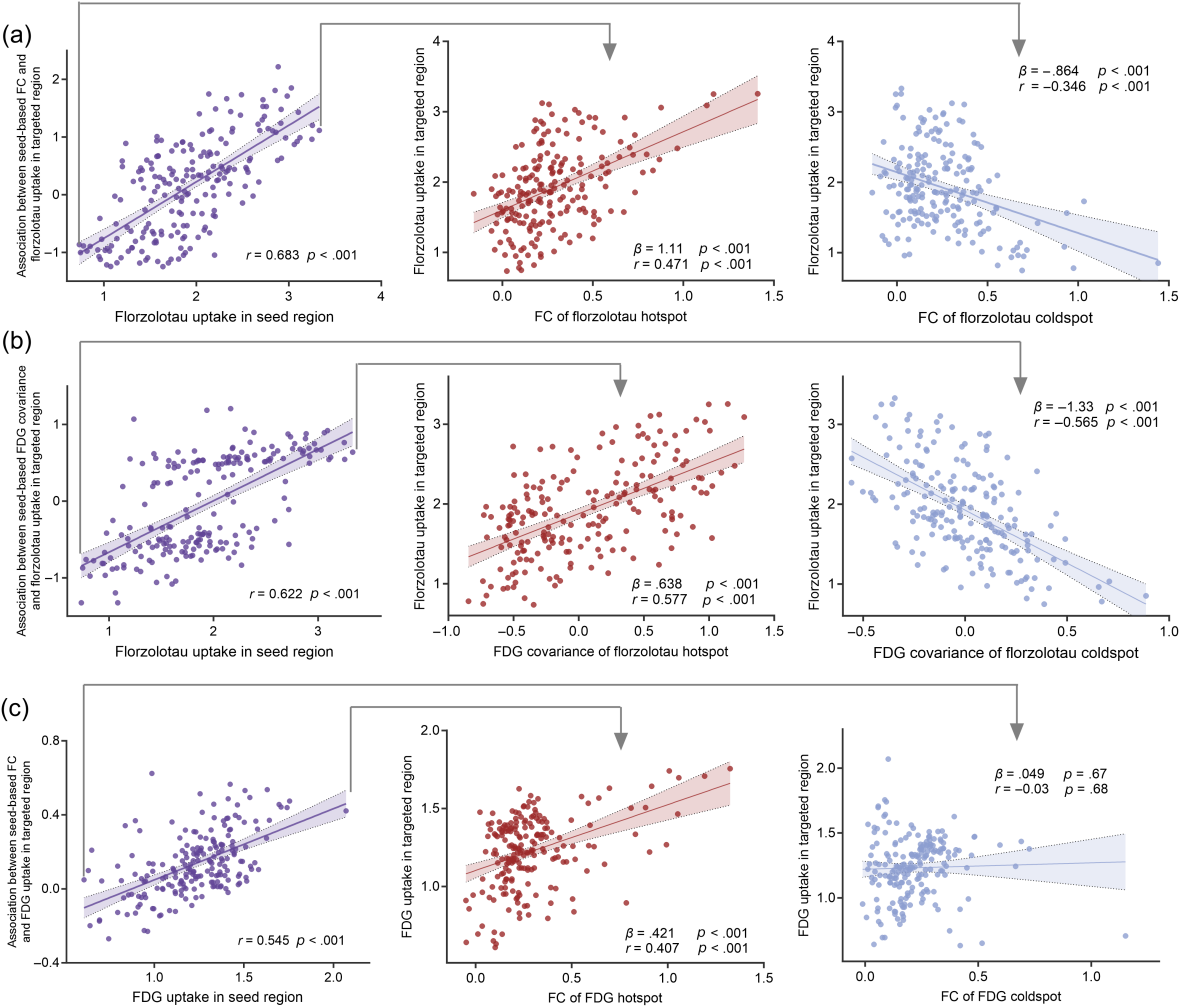
Association between Florzolotau uptake, fluorodeoxyglucose (FDG), and functional connectivity in AD group. (a) The association between Florzolotau uptake of a seed‐regions of interest (ROI) and the regression‐derived association between its' functional connectivity to target regions and Florzolotau in the respective target regions. (b) The association between Florzolotau uptake of a seed‐ROI and the regression‐derived association between its' FDG covariance (i.e., metabolic connectivity) to target regions and Florzolotau in the respective target regions. (c) The association between FDG uptake of a seed‐ROI and the regression‐derived association between its' functional connectivity to target regions and FDG in the respective target regions. FC, functional connectivity.

We next assessed whether the pathologic tau accumulation in a given seed ROI was associated with the tau uptake in closely connected regions of AD. As shown in Figure 4a, for seed regions with higher Florzolotau uptake, higher functional connectivity of a target region to this seed region was associated with higher Florzolotau uptake in the target region (i.e., positive *β*‐values in the regression). Vice versa, for seed regions with lower Florzolotau uptake, higher functional connectivity was associated with lower Florzolotau uptake in the target region (i.e., negative *β*‐values in the regression). We therefore conducted additional regression analyses to measure this potential tau spread pattern. There was a significant positive relationship between the Florzolotau SUVR in a seed region and their functional connectivity's predictive weight (corresponding *β*‐values for this given seed region) (*r* = 0.683, *p* < .001), which was consistently found in the relationship between the Florzolotau SUVR and their FDG covariance's predictive weight (*r* = 0.622, *p* < .001) and the relationship between the FDG SUVR and their functional connectivity's predictive weight (*r* = 0.545, *p* < .001), which remained after controlling for Euclidean distance. Again, the same statistical analyses applied to 500 null‐model connectivity or covariance, yielded shuffled *r*‐distributions of *r* = 0.046 ± 0.08 (Florzolotau and functional connectivity), *r* = 0.008 ± 0.12 (Florzolotau and FDG covariance), and *r* = 0.013 ± 0.09 (FDG and functional connectivity), yielding exact values of *p* < .001 when comparing the true *r*‐values against the null‐distributions. These results support the notion that the interaction mechanisms associated with tau spreading and downstream metabolism abnormality may be explained by functional connectivity.

In addition, the Florzolotau hotspot was located in the posterior cingulate cortex, whereas the Florzolotau coldspot was located in the left inferior frontal gyrus. Higher functional connectivity of the Florzolotau hotspot seed was associated with higher Florzolotau uptake in the target region (*β* = 1.1, *p* < .001; *r* = 0.471, *p* < .001) whereas higher functional connectivity of a target region with the Florzolotau coldspot was associated with lower Florzolotau uptake in the target region (*β* = −.864, *p* < .001; *r* = −0.346, *p* < .001). We also found a similar pattern of the relationship between Florzolotau and FDG covariance for Florzolotau hotspot (*β* = .638, *p* < .001; *r* = 0.577, *p* < .001) and coldspot (*β* = −1.33, *p* < .001; *r* = −0.565, *p* < .001), but the relationship between FDG uptake and functional connectivity was not significant for FDG coldspot (*β* = .049, *p* = .67; *r* = −0.03, *p* = .68). These above findings suggested that functional connectivity was not only associated with tau uptake, but also associated with FDG metabolism.

### 3.5 Mediation analyses

**FIGURE 5:**
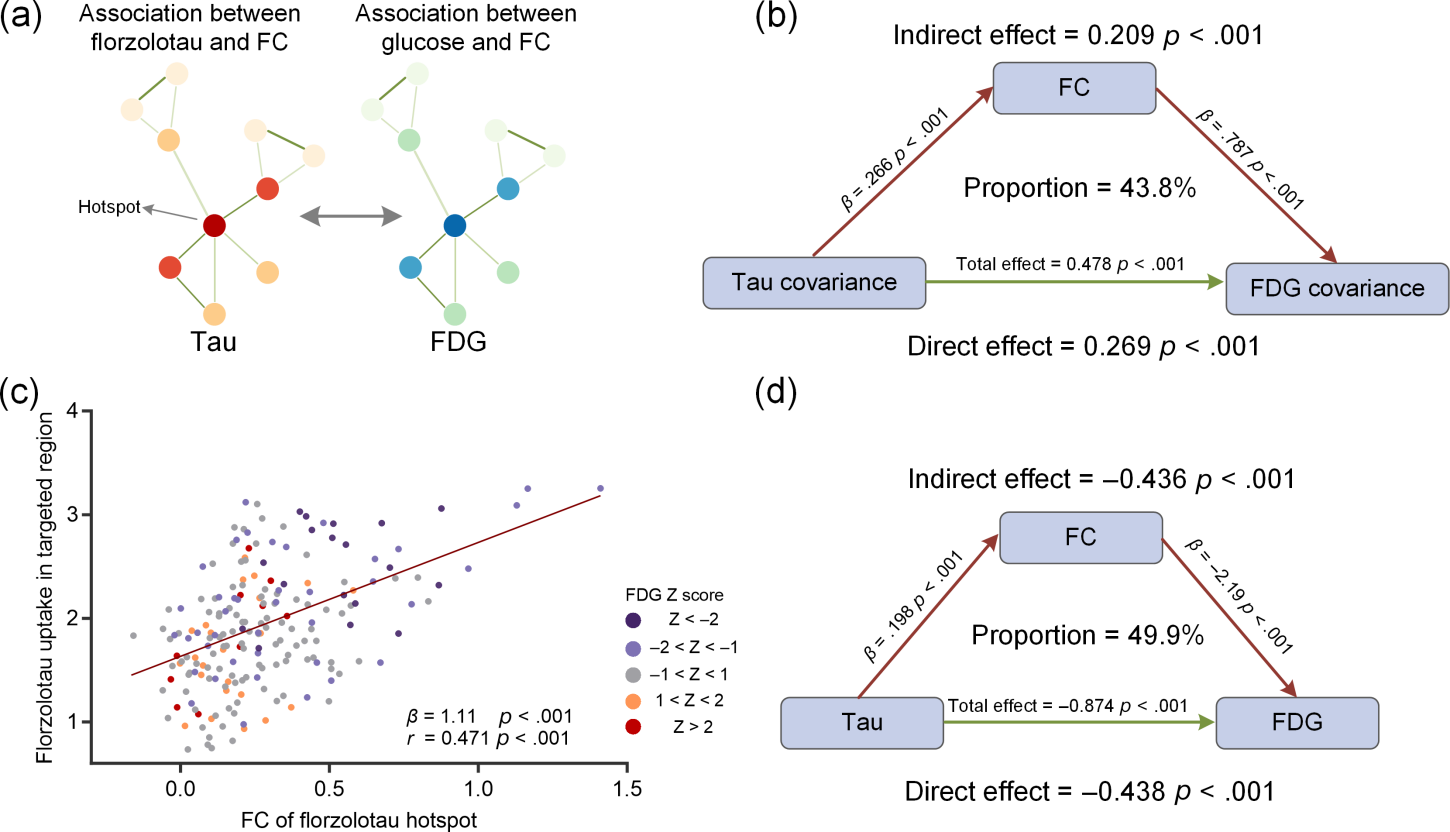
Mediation analyses with Florzolotau, fluorodeoxyglucose (FDG), and functional connectivity within the Florzolotau hotspot. (a) An artificial system based on Florzolotau spread with functional connectivity (FC) and corresponding FDG metabolism pattern with FC. (b) The relationship between tau covariance and FDG covariance measured by FC. (c) The linear regression analysis between functional connectivity of a target region with the Florzolotau hotspot and Florzolotau uptake in targeted region. Each dot in scatterplot was colored using FDG *Z*‐score, where blue color represents abnormally reduced metabolism in this brain region compared to normal subjects. (d) The relationship between Florzolotau and FDG measured by FC.

We further extended these associations by using the mediation model to test whether functional connectivity was a potential modulator of tau accumulation and glucose metabolism in AD (Figure 5a). As shown in Figure 5b, Florzolotau covariance was significantly associated with functional connectivity (*p* < .001) in the first equation, and also associated with FDG covariance (*p* < .001) in the second regression. In the last regression, when putting the functional connectivity and Florzolotau covariance simultaneously into the model, we found that the influences of Florzolotau covariance on FDG covariance remained but were significantly diminished. The effect was considered partial mediation, with 43.8% mediation proportion. Moreover, the similar mediation analysis was conducted in the Florzolotau hotspot region, as these regions with higher functional connectivity of targeted regions to this hotspot have similar glucose hypometabolism patterns (Figure 5c). Again, we found that the relationship between Florzolotau uptake and FDG metabolism was mediated mainly by functional connectivity, and the proportion of mediation is 49.9% (Figure 5d). These findings suggest that functional connectivity could at least partially modulate the influence of tau accumulation on glucose metabolism.

## 4 DISCUSSION

In this cross‐modal neuroimaging study, the major aim was to measure the association between tau accumulation and glucose metabolism and functional connectivity. Our results suggest that brain regions with higher tau accumulation tend to be connected to other regions with high tau accumulation through functional connectivity or metabolic connectivity (FDG covariance), and vice versa. Based on the above findings, we found that the association between tau accumulation and functional connectivity was closely linked with cascading glucose metabolism.

The association between functional connectivity and PET covariance of Florzolotau and FDG, and notably the significant relationships within seven functional networks, was a robust finding. These findings critically extend previous results, showing that higher functional connectivity is associated not only with higher Florzolotau covariance, but also with higher FDG covariance. The novelty of this study is to introduce FDG covariance (metabolic connectivity) to assess the relationship with tau spread. Most importantly, we found that there was a strong positive association between FDG covariance and Florzolotau covariance. Functional and metabolic connectivity of neural activity characterizes synchronicity in the blood oxygen level‐dependent and consistency in energy consumption of inter‐regional information transfer, respectively, which may suggest that the spread of pathologic tau is closely related to energy‐related connectivity. These findings are in line with previous similar studies showing that the spatial distribution of pathological tau exhibits patterns strongly reminiscent of brain functional networks (Hoenig et al., 2018; Jones et al., 2017; Vogel et al., 2019). The previous studies demonstrated that higher functional connectivity between brain regions is associated with similar tau‐level, functional brain architecture is associated with the rate of tau accumulation (Franzmeier et al., 2019; Franzmeier, Neitzel, et al., 2020). A recent study using tau accumulation and functional network measurements has indicated that functional hub regions are notably vulnerable to the accumulation of tau pathology (Cope et al., 2018). Altogether, these findings support the notion that higher functional or metabolic connectivity increases the susceptibility to the accumulation of pathological tau in analogous interconnected regions.

Our study further demonstrated that the posterior cingulate cortex Florzolotau hotspot exhibits a preferential connection to other regions with higher pathologic tau accumulation, as evidenced by both functional and metabolic connectivity analyses. In contrast, the inferior frontal gyrus Florzolotau coldspot exhibits a preferential connection to other regions with lower pathologic tau accumulation. Prior studies have demonstrated that the functional connectivity within rapidly accumulating tau regions potentially facilitates the propagation of tau pathology to their closely interconnected neighboring regions (Franzmeier et al., 2022; Franzmeier, Dewenter, et al., 2020; Franzmeier, Neitzel, et al., 2020). Our results were in line with previous evidence that functional or metabolic network‐forming regions show similar susceptibility for developing tau pathology (Grothe et al., 2018; Sepulcre et al., 2018). Multiple studies have previously reported that pathologic tau initially emerges in the transentorhinal cortex, progressing subsequently to the anterior hippocampus, adjacent limbic and temporal cortex, association isocortex, and ultimately affecting the primary sensory cortex in a sequential pattern (de Calignon et al., 2012; Schöll et al., 2016). Our findings of associations between tau covariance and functional/metabolic connectivity confirm that tau accumulation may be found preferentially along connected regions due to connectivity‐mediated tau spreading within DMN and DAN networks.

More, extensive evidence from causal mediation analyses suggests that functional connectivity partially modulates the influences of tau accumulation on FDG metabolism. This relationship between tau accumulation and downstream degeneration is in line with a recent compelling study showing that neurodegeneration (microglial activation) and tau accumulation spatially propagate in parallel following brain circuits predicted by postmortem series from the transentorhinal/entorhinal to sensorimotor cortices (Pascoal et al., 2021). Numerous in vitro and in vivo studies have demonstrated that elevated neuronal activity prompts the release of filamentous tau from neurons, initiating the process of micropinocytosis of pathological tau and aggregation in cascading neurons (DeVos et al., 2013; Pooler et al., 2013; Wu et al., 2016). These observations support the hypothesis of pathologic tau excitotoxic cascade, where the presence of pathologic tau excites neurons, resulting in overstimulation of connected neurons, subsequently compromising the trophic support to these neurons. Our findings support this hypothesis that neuronal connectivity patterns provide a better description of the spatial distribution of tau. The direct neurotoxic effects of tau disrupt neuronal glucose supply along with functional connectivity during its spreading. Consequently, this disruption triggers downstream events, resulting in disturbances in glucose metabolism within brain regions strongly associated with high tau accumulation. While the metabolism abnormality underlying tau spread may be explained by functional connectivity, there are likely additional contributing factors at play. Intrinsic molecular environment, shared genetic susceptibility, and directional flow of neuronal activity may achieve a more complete understanding of the interaction mechanisms underlying tau spreading.

Some limitations should be highlighted to appropriately interpret our results. The main limitation is that this study is cross‐sectional and enrolls AD patients with definite pathophysiology. We employed the mediation analysis in this cross‐sectional trial and further explored the relationship between functional connectivity and glucose metabolism underlying tau spreading, which revealed statistical associations but may not establish necessarily biological causations. Longitudinal patients within the whole AD continuum would be important and necessary. Further research should also conduct in‐depth and complete analyses of what role functional connectivity plays in the AT(N) research framework. In addition, we tested the associations between Florzolotau uptake and metabolic connectivity derived from group‐level data. To resolve this limitation, future studies will employ individual metabolic network analysis to obtain individual metabolic connectivity and further assess the interaction effects between tau and metabolism activity from an individual perspective. Lastly, NC enrolled in this study were younger than AD patients and had a small sample size, which may trigger metabolic differences caused by normal aging. Future studies will implement more precise age and sample size control measures to enhance the comparability of metabolic levels.

## 5 CONCLUSIONS

In summary, the current study demonstrates the significant associations between tau accumulation and functional connectivity and glucose metabolism in AD. These findings indicate the robust interaction between tau spreading through communicating connectivity and cascading glucose metabolism, emphasizing functional connectivity may partially modulate the influences of tau on FDG metabolism. Together, these findings support that connectivity is in some way involved in pathologic tau spreading and downstream glucose metabolism.

## CONFLICT OF INTEREST STATEMENT

The authors declare no competing interests.

## Supporting information


**Data S1.** Supporting information.

## Data Availability

Individual PET and MRI data requests in this study would be available after a reasonable request to the corresponding author, following a formal data sharing agreement.
