# Supplementary material for: Characterization of tau propagation pattern and cascading hypometabolism from functional connectivity in Alzheimer's disease
Source: Hum Brain Mapp. 2024 May 4;45(7):e26689. doi: 10.1002/hbm.26689 (PMC11069321; doi:10.1002/hbm.26689)
Supplement: Supplementary file 1 — Data S1. Supporting information. [file HBM-45-e26689-s001.docx]

Supplementary information of

**Characterization of tau propagation pattern and cascading hypometabolism from functional connectivity in Alzheimer’s disease**

Min Wang^1†^, Jiaying Lu^2†^, Ying Zhang^1^, Qi Zhang^1^, Luyao Wang^1^, Ping Wu^2^, Matthias Brendel^3^, Axel Rominger^4^, Kuangyu Shi^4,5^, Qianhua Zhao^6,7^, Jiehui Jiang^1*^, Chuantao Zuo^2,8*^

We used the Spearman correlation to measure the pairwise correlation value and further obtain a single 200× 200 sized covariance in Florzolotau or FDG matrix. Otherwise, an additional covariance in tau/FDG matrix controlled for age, sex, and education was obtained using partial correlation for each ROI pair. There were no obvious differences between PET covariances and covariate-controlled PET covariances (Fig.S1). We then re-calculated the associations between functional connectivity and covariate-controlled PET covariance (Florzolotau and FDG) in whole-brain and network-specific manners. As shown in Fig.S2, the associations between functional connectivity and PET covariances remained consistent when controlling the assessment of PET covariances for age, sex, education.


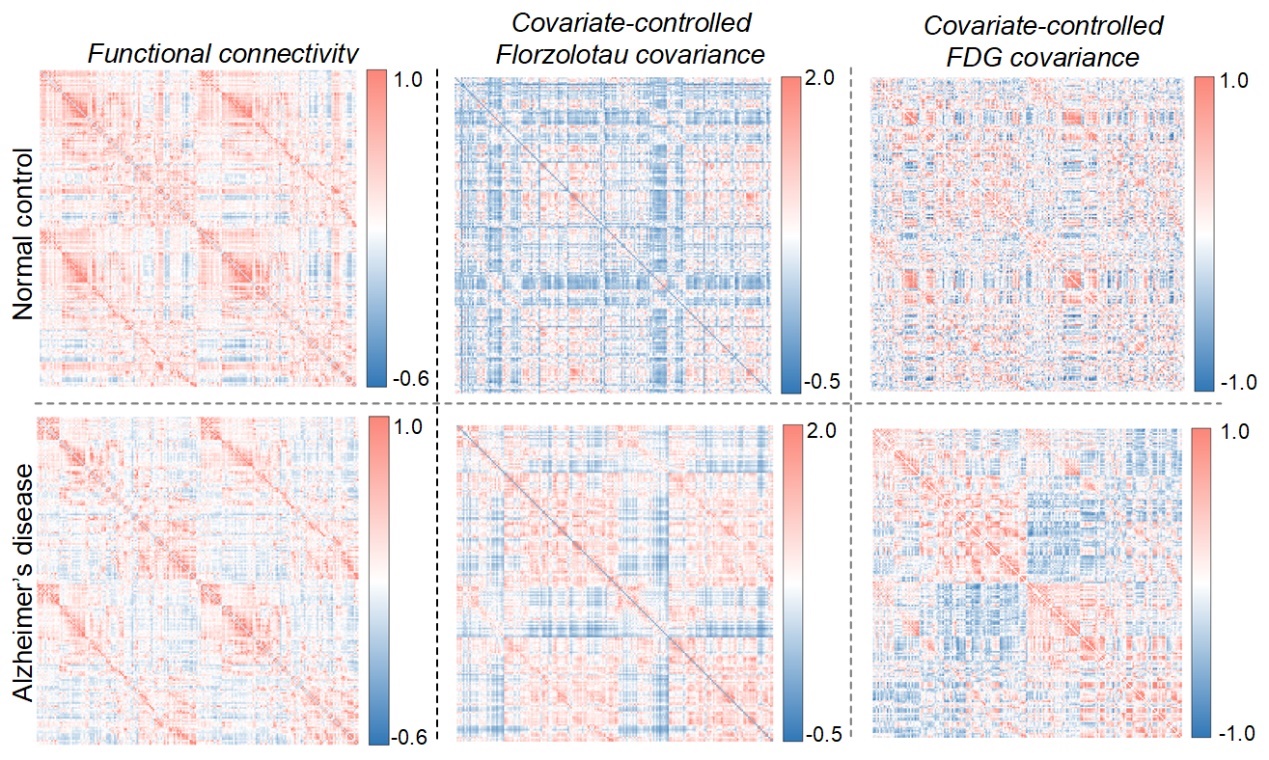


Figure S1. Average functional connectivity and covariance of Florzolotau and FDG matrices controlled for age, sex, and education in normal control and Alzheimer’s disease groups. All correlation values in 200 × 200 matrix was Fisher-z transformed.


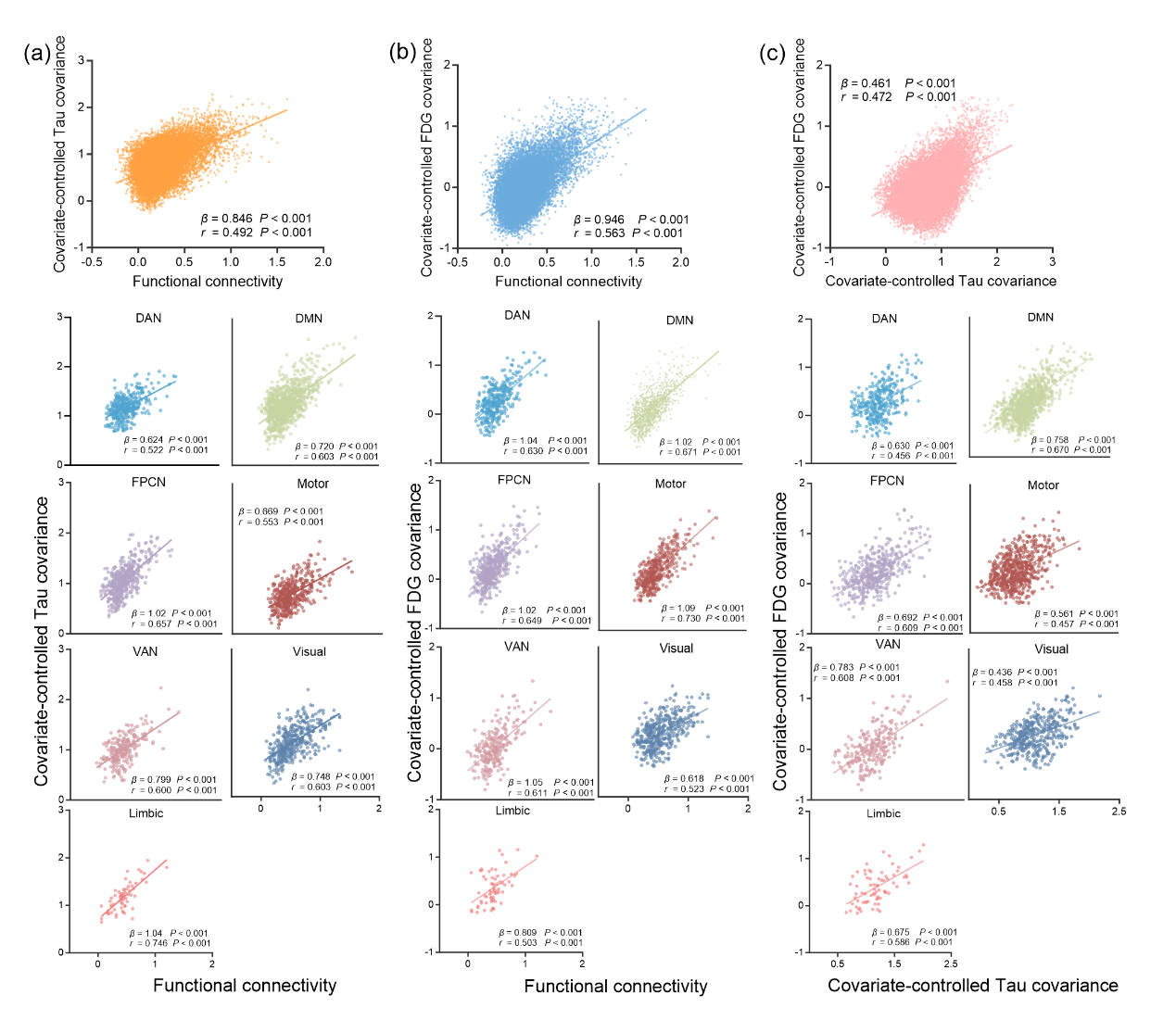


Figure S2. Associations between functional connectivity and covariate-controlled PET covariance. Scatterplots showing the associations between functional connectivity and Florzolotau covariance (a), FDG covariance (b), and Florzolotau covariance and FDG covariance (c).


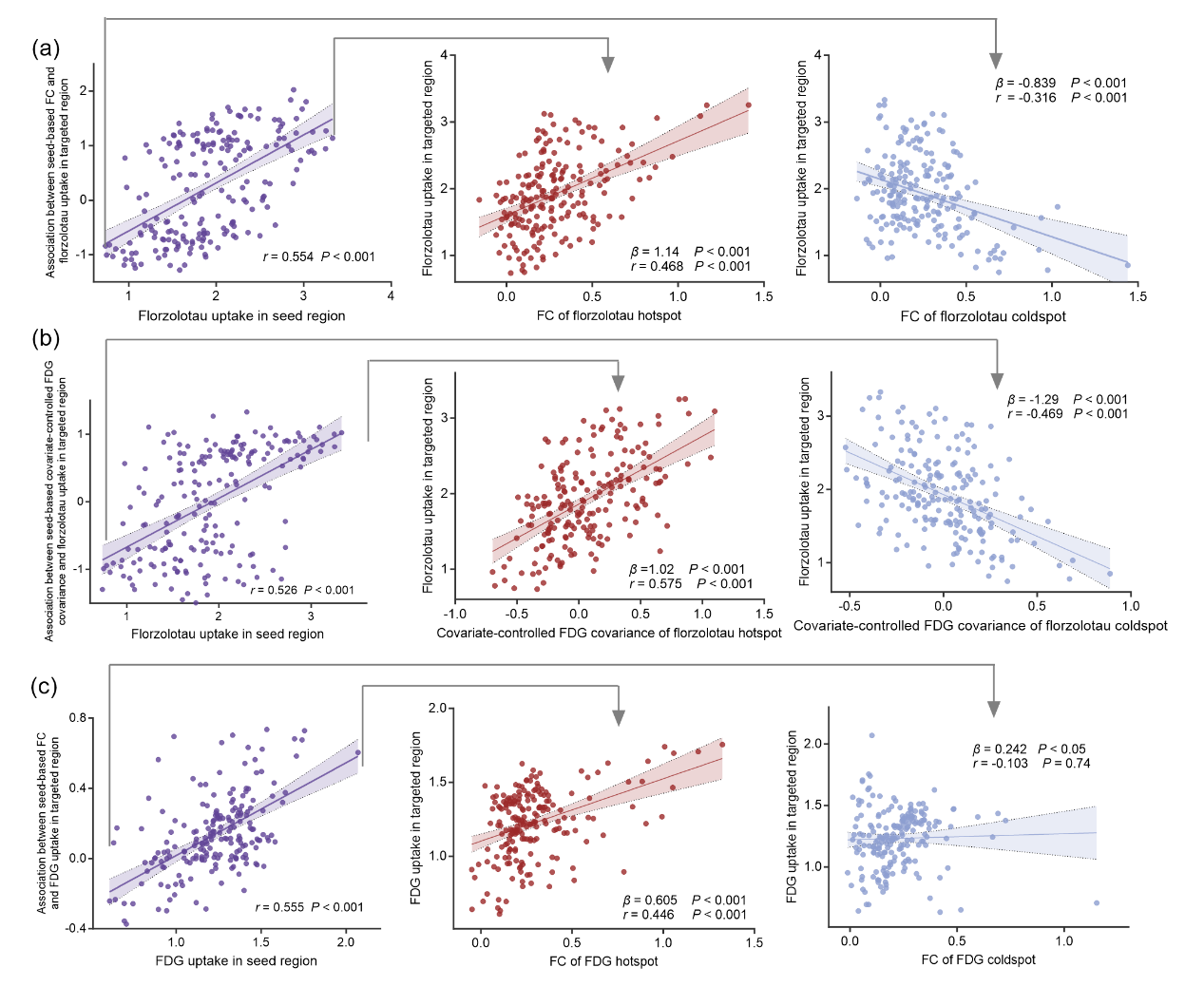


**Figure S3**. Association between Florzolotau uptake, FDG, and functional connectivity in AD group while additionally controlling the regression model for Euclidean distance between each ROI pair. (a) The association between Florzolotau uptake of a seed-ROI and the regression-derived association between its’ functional connectivity to target regions and Florzolotau in the respective target regions. (b) The association between Florzolotau uptake of a seed-ROI and the regression-derived association between its’ covariate-controlled FDG covariance (i.e. metabolic connectivity) to target regions and Florzolotau in the respective target regions. (c) The association between FDG uptake of a seed-ROI and the regression-derived association between its’ functional connectivity to target regions and FDG in the respective target regions.
